# Cdh4 Down-Regulation Impairs in Vivo Infiltration and Malignancy in Patients Derived Glioblastoma Cells

**DOI:** 10.3390/ijms20164028

**Published:** 2019-08-18

**Authors:** Davide Ceresa, Francesco Alessandrini, Lorenzo Bosio, Daniela Marubbi, Daniele Reverberi, Paolo Malatesta, Irene Appolloni

**Affiliations:** 1 U.O. Oncologia Cellulare, Ospedale Policlinico San Martino, 16132 Genova, Italy; 2 Department of experimental medicine (DIMES), University of Genova, 16132 Genova, Italy; 3 U.O. Molecular Pathology, Ospedale Policlinico San Martino, 16132 Genova, Italy

**Keywords:** brain tumor, contact inhibition, R-cadherin, proliferation, migration, adherent junctions, cell polarity

## Abstract

The high invasive phenotype of glioblastoma is one of the main causes of therapy inefficacy and tumor relapse. Cell adhesion molecules of the cadherin family are involved in cell migration and are known as master regulators of epithelial tumor invasiveness, but their role in glioblastoma is less understood. In particular, we recently demonstrated, in the syngeneic murine model, the occurrence of a previously undescribed cadherin switch between Cdh2 and Cdh4 during gliomagenesis, which is necessary for the acquisition of the highly infiltrative and tumorigenic phenotype of these cells. In the present study, we tested the role of Cdh4 in human gliomas. Our results on patient-derived glioma cells demonstrate a positive correlation between Cdh4 expression levels and the loss of cell–cell contact inhibition of proliferation controls that allows cells to proliferate over confluence. Moreover, the silencing of Cdh4 by artificial microRNAs induced a decrease in the infiltrative ability of human glioma cells both in vitro and in vivo. More strikingly, Cdh4 silencing induced an impairment of the tumorigenic potential of these cells after orthotopic transplantation in immunodeficient mice. Overall, we conclude that in human glioblastoma, Cdh4 can also actively contribute in regulating cell invasiveness and malignancy.

## 1. Introduction

Gliomas are primary tumors of the central nervous system. The most malignant form, named glioblastoma, has a dismal prognosis. Even with the best currently available treatment, the median survival time from diagnosis is 15 months [1].

Besides the typical radio- and chemo-resistance, the high infiltrative phenotype of glioma cells in the brain parenchyma is a crucial feature of gliomas that makes surgical resection ineffective and relapses very common [2]. In this context, a better understanding of the mechanisms underlying cell infiltration is essential to defeat this tumor and many studies contribute in dissecting the molecular pathways underlying this process [3,4]. One of the analyzed mechanisms, typical of metastatic epithelial tumors, is the epithelial to mesenchymal transition (EMT) [5]. In this process, the activation of signaling pathways such as TGF-β or Wnt/β-catenin induces a switch from E-Cadherin (Cdh1) to N-cadherin (Cdh2) that loosens the adherent junctions and promotes cell migration [3,6]. In gliomas, the role of EMT is still debated. Many studies show the upregulation in gliomas of genes typically involved in EMT [4,7,8,9,10], but the role of cadherins is still under analysis. On the contrary of other epithelial tissues, neural cells normally express Cdh2 instead of Cdh1 [11,12,13]. Moreover, while some data indicate that Cdh2 expression correlates with tumor grade or invasiveness [14,15,16] other studies reported opposite results [17,18,19]. Camand and collaborators explained this contradiction with a difference between Cdh2 mRNA and protein expression level, with the former highly expressed and the latter scarcely expressed in glioma samples. The same authors further demonstrated that Cdh2 downregulation increases cell migration of both normal neural cells and glioma cells [17] showing an anti-migratory role for Cdh2 in neural tissue.

In line with the previous data we recently demonstrated, for the first time, the existence of an alternative cadherin switch during gliomagenesis taking place between Cdh2 and Cdh4 [20], a classical cadherin is expressed in the neural tissue both during development and adult life [13,21,22,23,24,25,26]. This event, that takes place at the adherent junctions of malignant cells, allows glioma cells overcoming the mechanisms of cell–cell inhibition of proliferation (CIP) and migration (CIM), and makes them proliferate in an uncontrolled manner and infiltrate the brain parenchyma. The results showed that Cdh4 is necessary for glioma invasion and for the maintenance of the tumorigenic potential of these glioma cells. All these data clearly show that a cadherin switch plays a crucial role in regulating proliferation and cell migration in glioma in a similar way to what has been demonstrated for epithelial tumors, although involving slightly different molecular players. Moreover, Cdh4 expression seems important for tumorigenesis in different tumor types. However, the role of this molecule has not been fully clarified yet, since in some tumors it promotes malignancy while in other it acts as a tumor suppressor [27,28,29,30,31,32].

In the present study, to elucidate the role of Cdh4 on glioma cells migration, we extended our analysis in patient-derived glioblastoma initiating cells (GIC). Our data suggest that Cdh4 is a useful prognostic marker for glioma and, although down-regulation is not sufficient to fully inhibit glioma invasion as was in the murine model, we showed a partial rescue of CIM mechanisms in vitro and a reduced migratory activity in vivo. Moreover, Cdh4 silencing reduces the tumorigenic potential of GIC cells in vivo.

## 2. Results

### 2.1. Cdh4 Is Heterogeneously Expressed by Human Gliomas and Highlights Tumors Ability to Bypass the Cell–Cell Contact Inhibition of Proliferation

**Figure 1:**
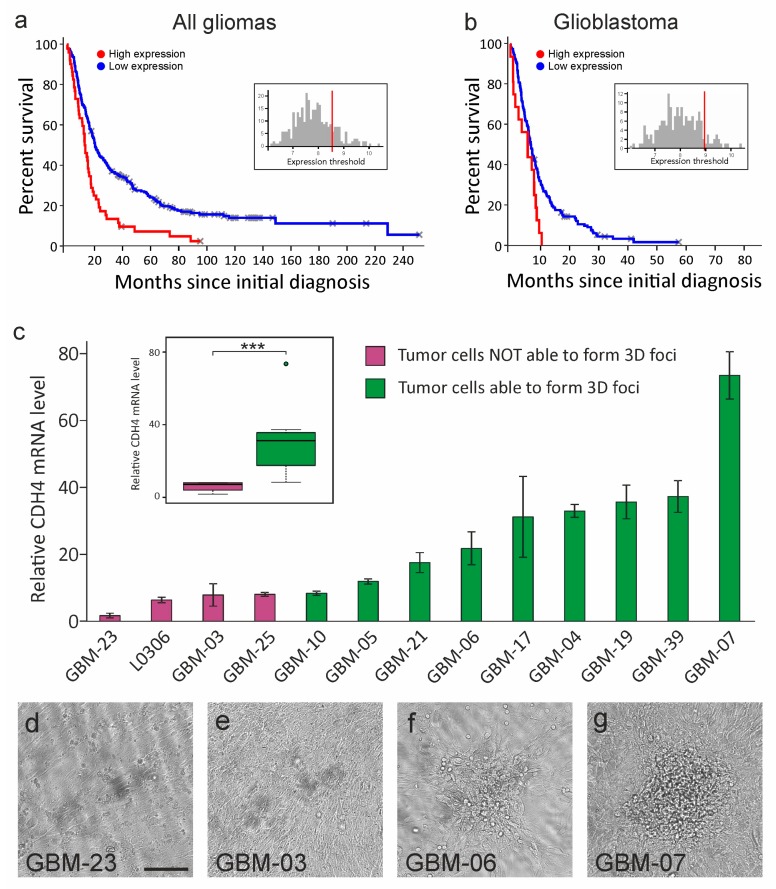
Cdh4 expression in human gliomas. (**a**,**b**) Kaplan–Meier curves of all glioma (**a**) or glioblastoma (**b**) patients with low or high Cdh4 expression level. The inset shows the frequency plot of Cdh4 expression level in the analyzed tumors. Threshold was chosen to group in the high expression pool tumors with a Cdh4 expression level higher respect to healthy tissue. (**c**) The histogram shows the quantification by quantitative PCR of Cdh4 mRNA level in different human glioblastoma initiating cells (GIC) cultures normalized to GBM-23, which have the lowest Cdh4 expression level. Purple bars represent GICs whose proliferation is inhibited by cell–cell contact, while green bars represent GICs able to proliferate over cell confluence. The barplot in the inset shows the differential Cdh4 expression levels between these two groups of GICs. (**d**–**g**) Bright field micrographs representing over confluence GIC cultures. Scale bar: 500 µm. *** *p* < 0.001.

Our previous work demonstrated that Cdh4 is necessary for the maintenance of the full malignant phenotype in a murine model of glioma [20]. Moreover, a metadata analysis on public datasets showed that Cdh4 expression level correlates with a shorter patients’ survival, suggesting for this protein an oncogenic role also in human gliomas. An updated analysis on the public repository Rembrandt (Betastasis, available online: http://www.betastasis.com/glioma/rembrandt) confirmed the prognostic value of Cdh4 expression level on both all glioma subtypes (*n*= 329; *p* < 0.001) and glioblastoma alone (*n* = 178; *p* < 0.02; Figure 1a,b).

We therefore analyzed, by quantitative PCR, the expression level of *Cdh4* gene on 12 different patient-derived GIC cultures. As shown in Figure 1c, we noticed a large heterogeneity in the Cdh4 mRNA levels that encompass about two orders of magnitude. A tendency similar to that observed in Figure 1b was noticeable in the survival of the patients in this set. Stratifying the patients with a known survival time based on Cdh4 expression, we observed a shorter overall survival time for the high Cdh4 expressing group that have a median survival time of 14.2 months versus 9.6 months of low Cdh4 expressing group (Figure S1).

Considering our previous data on the murine glioma model showing the role of Cdh4 in overriding the mechanism of CIP, we tested the ability of human GIC cultures to proliferate over confluence forming 3D foci. As shown by the color code in the histogram in Figure 1c and by bright-field micrographs in Figure 1d–g, there is a threshold level of Cdh4 expression beyond which GIC cultures acquire the ability to grow over confluence in vitro. Moreover, dividing the analyzed GICs on the basis of their ability to form 3D foci we noticed a significant differential Cdh4 expression level between the two groups (*p* < 0.01). These data suggest that, similarly to what observed in the murine model, Cdh4 expression can allow cells to contrast CIP in human GIC.

**Figure 2:**
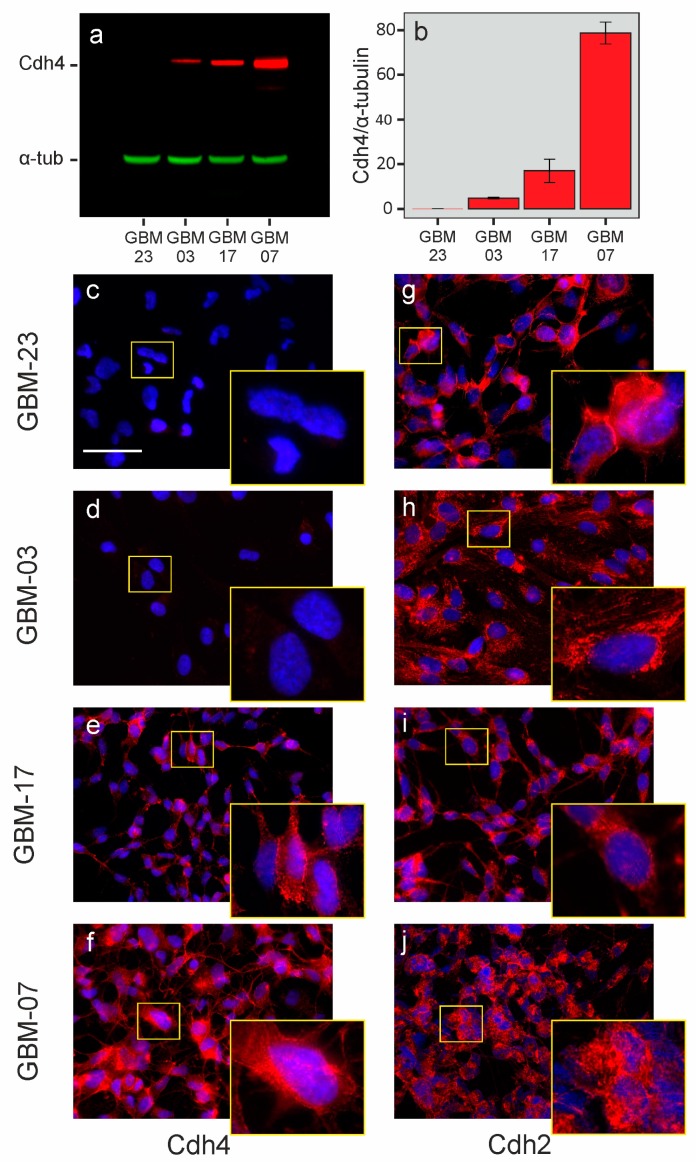
(**a**,**b**) Quantification of Cdh4 protein expression by Western blot of different GIC cultures. (**c**–**j**) Representative immunofluorescence stainings of different human GIC cultures with anti-Cdh4 (**c**–**f**) and anti-Cdh2 (**g**–**j**) antibodies in red and Hoechst for nuclei staining in blue. Scale bar: 50 µm.

We previously demonstrated that Cdh4 can compete with Cdh2 for membrane localization in mouse glioma cells, inducing a cadherin switch similar to that described in the EMT process occurring during epithelial tumor progression. Therefore, we performed western blot analysis and immunofluorescence staining on a subset of GIC cultures to confirm Cdh4 expression data at protein level and to investigate the localization of Cdh4 and Cdh2. We noticed that, in the analyzed GIC cultures, Cdh4 protein levels correlate to mRNA levels (Figure 2a–f), and that Cdh2 is predominantly localized in the perinuclear region (Figure 2g–j). Only GBM23, the glioma culture with the lowest Cdh4 expression level, shows Cdh2 protein localization in the cell-cell junction region forming septa between adjacent cells (Figure 2g).

### 2.2. The Silencing of Cdh4 Is Not Sufficient to Restore Cell–Cell Contact Inhibition of Proliferation

**Figure 3:**
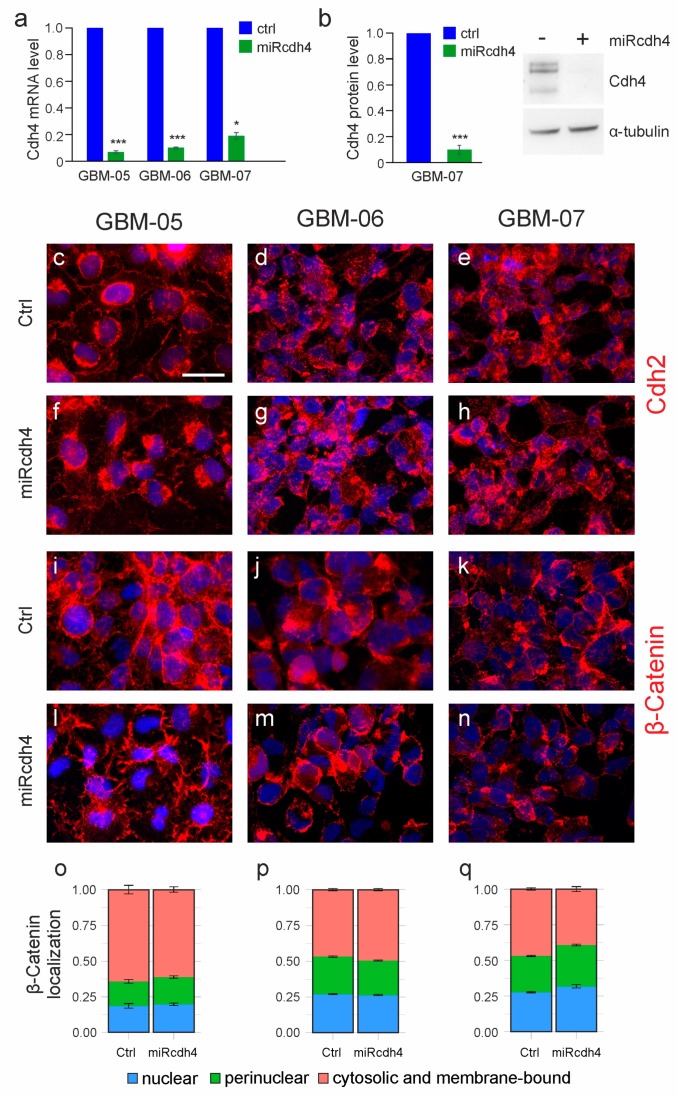
Effect of the miRcdh4 transduction in GICs. (**a**,**b**) Quantification of Cdh4 mRNA expression by quantitative PCR (**a**) or protein expression by western blot (**b**) of Cdh4 silenced GICs compared to control transduced GIC cultures. (**c**–**n**) Representative immunofluorescence stainings with anti-Cdh2 (**c**–**h**) or anti-β-catenin (**i**–**n**) antibodies of different Cdh4 silenced or control transduced human GIC cultures. Nuclei are showed in blue using Hoechst. Scale bar: 25 µm. (**o**–**q**) Subcellular localization of β-catenin immunoreactivity in the different GIC cultures upon miRcdh4 or control transduction, assessed by image analysis. Values are reported as fraction of total β-catenin signal. * *p* < 0.05, *** *p* < 0.001.

All these data suggest that Cdh4 could have a role in the acquisition of a malignant phenotype in gliomas. To assess this possibility, we downregulated Cdh4 in a subset of GIC cultures. In particular, we chose three GICs between the group of Cdh4 high-expressing gliomas: GBM-05, which has the lowest expression level in the group, GBM-06, which has an intermediate value and GBM-07, which has the highest expression level (Figure 1c). Cdh4 silencing was performed by transducing GIC cultures with a high titer pool of six different microRNA-expressing retroviral particles (hereinafter named miRcdh4) to allow multiple infection per cell and to increase the efficiency of Cdh4 downregulation due to the simultaneous targeting of different Cdh4 mRNA regions. As shown in Figure 3a, miRcdh4 transduction induced a strong downregulation of Cdh4 mRNA in all the three GIC cultures (Cdh4 mRNA was 7 ± 0.6% of the control in GBM-05, 10 ± 0.3% of the control in GBM-06 and 19 ± 1.8% of the control in GBM-07). GBM-07, which showed the lowest silencing efficiency by quantitative PCR, was also analyzed by western blot. The analysis revealed that Cdh4 protein levels dropped to 10 ± 3% compared to control transduced cells (Figure 3b).

The analysis of the expression levels of Cdh2 by immunofluorescence staining revealed no differences in its localization between Cdh4-silenced and control cultures (Figure 3c–h), with the protein expressed in the perinuclear area and at a slighter level in the cell-cell contact region. In the epithelial tumor EMT, the cadherin switch at the adherent junctions is correlated with a nuclear shuttling of β-catenin [33]. We therefore assessed the localization of β-catenin in GIC cultures. As shown in Figure 3i–n and quantified in Figure 3o–q, β-catenin has mainly a cytoplasmic localization in both Cdh4 silenced and control GICs, suggesting that the β-catenin pathway is not involved in these gliomas [34,35] as we showed also for the murine model [20]. Moreover, we noticed a tendency in the modulation of ERK and p27 pathways (Figure S2) in line with our previous data on mouse glioma cells. Cdh4 silencing is associated with a lower level of ERK phosphorylation (Figure S2a,b) and an increased level of the cell cycle inhibitor p27 (Figure S2c,d). The AKT (Protein Kinase B) pathway is not activated in our GIC cultures since the phosphorylated form of AKT is almost absent in these cells and it is not influenced by Cdh4 modulation (Figure S2a) as for the murine gliomas [20].

**Figure 4:**
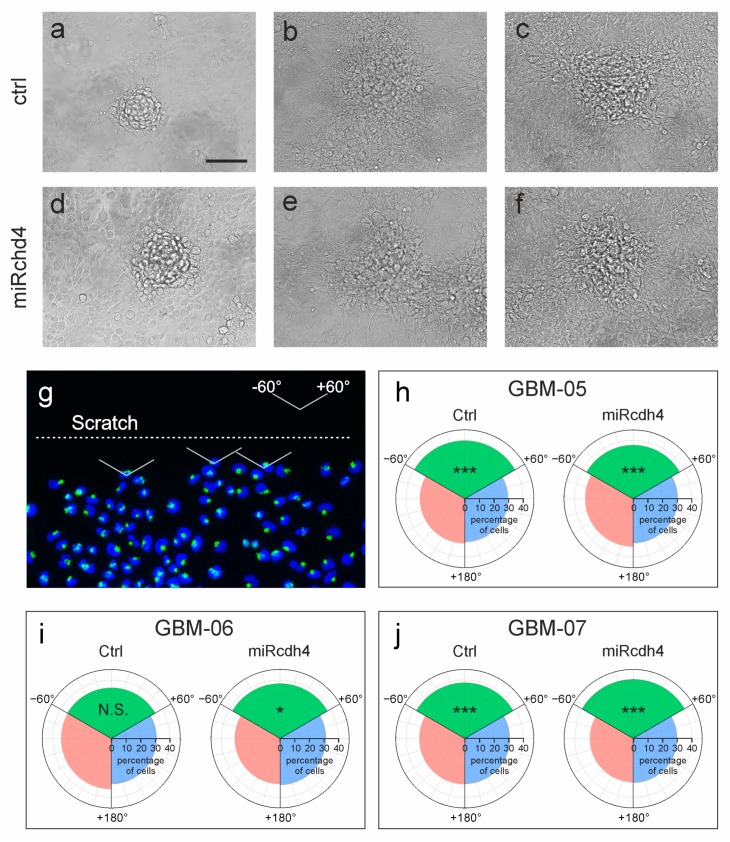
Effect of Cdh4 silencing on contact inhibition. (**a**–**f**) Bright field micrographs showing over confluence cultures of different Cdh4 silenced or control transduced human GICs. Scale bar: 500 µm. (**g**–**j**) Analysis of cell polarization of Cdh4 silenced GICs. (**g**) Example of the cell polarization analysis. Golgi apparatus was shown in green with anti-GM-130 antibody and nuclei in blue by Hoechst staining. (**h**–**j**) Polar histograms represent the migration directions of the cells close to the rim scratch. Cells with migration direction between −60° and +60° were considered polarized toward the scratch (green sector). *χ*^2^ test was used to assess whether the cells of each population were biased toward the scratch. * *p* < 0.05,*** *p* < 0.001.

To analyze whether Cdh4 silencing is sufficient to restore CIP, we cultured the three downregulated GIC cultures over confluence and found that Cdh4 downregulation does not impair the ability of glioma cells to growth over confluence forming 3D foci in vitro (Figure 4a–f).

### 2.3. The Silencing of Cdh4 Reduces Tumor Infiltration and Proliferation

Besides the acquisition of a high proliferation aptitude, another important feature of malignancy is the ability of tumor cell to infiltrate the surrounding tissue overriding the mechanisms of CIM and migrating notwithstanding the presence of other cells. To test whether Cdh4 has a role in promoting glioma cells infiltration, we performed an in vitro wound healing assay scratching confluent cultures of both control and Cdh4 silenced GICs. After 8 h, we analyzed whether glioma cells have a preference in migrating towards the scratch or whether they are able to migrate in any direction regardless the presence of other cells. Cell polarization was analyzed by evaluating, in the cells adjacent to the scratch, the relative position of the cell nucleus and the Golgi apparatus, by immunofluorescence staining with anti-GM130 antibody. The direction of the vector starting in the nucleus center of mass and pointing to the Golgi apparatus was considered as the migration direction of each cell [36] (Figure 4g). Directions were referred as 0° when perpendicular to the scratch. Cells with a migration direction between −60° and +60° were considered as polarized toward the scratch.

Our analysis showed that GBM-05 cells were already polarized toward the scratch (*n* = 3, ncells = 1465, *χ*^2^: *p* < 0.001), revealing a preserved CIM control (typically correlated with limited infiltrative potential). The downregulation of Cdh4 in this culture did not alter this tendency (*n* = 4, ncells = 2739, *χ*^2^: *p* < 0.001; Figure 4h). GBM-06 did not show polarization (i.e., GBM-06 cells migrate indifferently in any direction; *n* = 3, ncells = 787, *χ*^2^: *p* > 0.4, with a power > 80%, for effect size = 0.1). Cdh4 silencing in these cells dramatically change this situation, restoring CIM control as shown by the acquisition of cell polarization towards the scratch (*n* = 3, ncells = 489, *χ*^2^: *p* < 0.05; Figure 4i). GBM-07 cells, similarly to GBM-05, appeared polarized towards the scratch both in the control (*n* = 3, ncells = 3262, *χ*^2^: *p* < 0.001) and in the Cdh4 silenced culture (*n* = 3, ncells = 3144, *χ*^2^: *p* < 0.001; Figure 4j). These data show that Cdh4 silencing can restore CIM control in cells that have lost it suggesting that Cdh4 might contribute to tumor cells infiltration. We therefore checked whether Cdh4 is necessary for glioma cells infiltration in the brain parenchyma.

We injected in NOD/SCID murine brains a mixed population of control GIC, expressing only DsRed, together with Cdh4-silenced GIC, expressing the 6 synthetic microRNAs against Cdh4 and GFP reporter. We then compared their migratory ability 18–21 days after the transplant. We noticed that GBM-05 and GBM-06 does not migrate massively at these time points, neither 30 days after cell transplantation. Only few cells have left the injection site and are still in the nearby (data not shown) so we could not analyze the effect of Cdh4 downregulation on these two gliomas in vivo.

**Figure 5:**
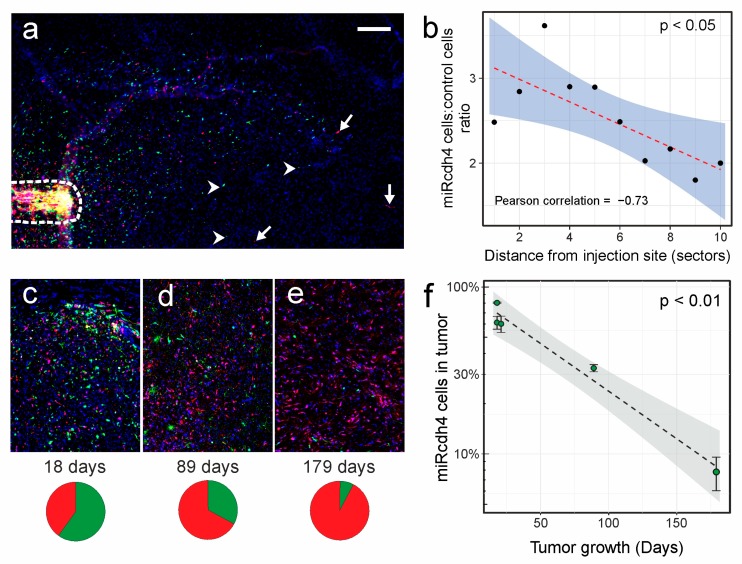
In vivo analysis of migration and proliferation of Cdh4 silenced GBM-07 cells. (**a**) Representative brain section near the injection site after 18 days from the transplant of Cdh4 silenced (green) or control (red) GBM-07 cells. Arrowheads point representative Cdh4 silenced cells; arrows point examples of control transduced cells; the border of the injection site is marked by a dashed line. Cell nuclei (blue) are counterstained with Hoechst. (**b**) Negative correlation between the enrichment of Cdh4 silenced cells and their distance from the injection site. For each section, all distances have been divided in 10 sectors of equal size to enable a cumulative analysis. Statistical analysis was performed by Pearson correlation test. (**c**–**e**) Representative images of tumor sections after 18 (**c**), 89 (**d**), and 179 (**e**) days after transplant. (**f**) Proportion of Cdh4 silenced cells in the total tumoral mass markedly decreases over time. Each point represents a different tumor and error bars show intra-tumoral variability. Statistical analysis was performed by the Pearson correlation test. Scale bar: 500 µm (**a**), 200 µm(**c**–**e**).

GBM-07 has a high migratory phenotype and both control and Cdh4 silenced cells can infiltrate the brain parenchyma (Figure 5a) with some of them reaching the contralateral hemisphere. To check whether Cdh4-silenced GICs have a reduction in their migratory ability respect to control cells, we performed a quantitative analysis on 4 injected mouse brains measuring the distance from the injection site for each cell outside the needle track. We noticed, between the analyzed brain sections, a large heterogeneity in the maximum distance walked by cells from the injection site that limits data comparison between different sections. Therefore, we normalized all cells’ routes dividing by 10 the maximum cell distance found per section and generating discrete distance estimators (bins) comparable across sections. The ratio between the number of microRNA transduced and control cells per bin decreases with cells distance unveiling a lowering of the infiltrative ability of the Cdh4 silenced population respect to control transduced GICs (ncells= 3879 miRNA, 1406 ctrl; Pearson correlation: 0.78, *p* < 0.01; Figure 5b). This result corroborates the in vitro data about a role for Cdh4 in disrupting the CIM control system and in favoring glioma infiltration in the brain parenchyma. However, our data showed that in human gliomas Cdh4 silencing is not sufficient to completely inhibit the infiltrative phenotype and, possibly, other mechanisms contribute to this process.

To investigate the effect of Cdh4 silencing in the in vivo proliferation ability, we orthotopically injected 2 mice with the same mixed population of control and Cdh4-silenced GIC of the previous experiment, and we analyzed them at later times (89 and 179 days, respectively). We therefore measured the percentage of Cdh4-silenced GIC in the total tumoral cells in these two late tumors and in the earlier sections analyzed 18–21 days after injection. The percentage of Cdh4-silenced GIC dramatically decreases with time (Figure 5c–f; Pearson correlation test *p* < 0.01) from 67 ± 7% (18–21 days) to 33 ± 2% (89 days) and lastly to 8 ± 2% (179 days). This result suggests that GBM-07 is addicted to Cdh4 expression to maintain its full malignancy.

## 3. Discussion

Cdh4 is typically expressed in the nervous system during both development and adulthood [13,21,22,23,24,25,26,37]. Many studies showed its central role in regulating neuronal progenitors migration [38,39] especially during radial migration in the cortical plate along radial glia cells [26,40,41]. This cells/neurites guidance activity seems at least in part due to the combinatorial cadherins expression that during development generates a molecular code allowing cell sorting and fasciculation based on preferential cadherin homophilic dimerization [21,42,43]. The Cdh4 role in cancer is still less clear since in some studies it seems to have oncogenic properties [29,32] maintaining stemness features and increasing tumor cell migration and malignancy, while in other cases it acts as an oncosuppressor since its dowregulation increases malignancy in different tumor types [27,28,30,31]. These contrasting results can be due to different functions performed by Cdh4 in different tumor types. It has been shown for other cadherins a context-depending activity, since both intracellular and extracellular matrix (ECM) molecules can highly influence cadherin adhesive properties modifying the strength of both cell-cell and cell-matrix junctions [44,45,46,47]. A particular context can for instance promote a more pronounced migratory ability for Cdh1 expressing cells without the need of a cadherin switch [48,49].

In a previous work on a glioma model in mouse, we demonstrated that Cdh4 is necessary to sustain cell infiltration and proliferation in vitro and in vivo by overcoming both CIP and CIM mechanisms. This characteristic is typical during the EMT process often occurring in tumor malignancy where, thanks to a switch between Cdh1 and Cdh2, tumor cells loosen cell-cell adhesivity [50]. We demonstrated for the first time that during gliomagenesis an alternative cadherin switch occurs between Cdh2 and Cdh4 [20].

In this work we focused on the role of Cdh4 in human glioblastoma. Altogether our data demonstrated that, in such a context, Cdh4 could be a useful prognostic marker. Its expression is heterogeneous within glioma patients and correlates with a shorter survival time in glioma patients and with the acquisition, by glioma cells, of high malignant features as the loss of cell-cell contact inhibition mechanisms. In line with our previous results on murine cells, we noticed a switch on the cell membranes between Cdh2 and Cdh4. In GBM-23, where Cdh4 is not expressed (Figure 2a–c), Cdh2 is localized at the cell-cell junctions often forming visible septa (Figure 2g) while, there is a delocalization in Cdh4 expressing tumors (Figure 2h–j). Unlike in mouse however, Cdh4 downregulation does not revert this phenotype. Several reasons can explain this result as the presence of other molecules able to compensate Cdh4 activity. Alternatively, post-translational modifications on Cdh2 (as phosphorylation) could prevent its recycling on the membrane [51,52,53,54]. Additionally, there is the possibility that the residual Cdh4 levels upon miRcdh4 transduction are still sufficient to induce Cdh2 delocalization.

Our results showed that, although Cdh4 silenced glioma cells can proliferate and infiltrate the brain parenchyma, these abilities result highly impaired. In an in vivo competition assay, Cdh4 downregulated cells migrate less than control cells and six months after orthotopic transplantation they represent less than 10% of the initial mixed cell population. We wonder whether the observed differences in the migratory behavior could have alternative explanations and, in particular, could depend on the known difference in cell proliferation. Although proliferation cannot explain, by itself, differences in migration, and faster proliferative activity might lead to a more abundant cell population and the different population size might, in turn, translate in an artefactual difference in migration. In fact, for purely statistical reasons, a larger cell population will have maximal distance from the injection site larger than the maximal distance reached by cells from smaller population, even if they have the same infiltrative abilities. In our experiments, however, Cdh4 cells (which show lower proliferation rate) were injected in a larger amount and, more importantly, they were still in larger amount at the time of the analysis (18–21 days post injection).

The Cdh4-dependent inhibition of CIP and CIM mechanisms allowing cells to proliferate and migrate notwithstanding the presence of other cells, can be explained by a Cdh-4 dependent loosening of the adherent junctions, as in the classical EMT, where a cadherin with a reduced adhesive strength (Cdh2) substitutes a cadherin with a higher adhesive strength [55,56]. Further data on Cdh4 adhesion strength will help to clarify this point. An alternative explanation could be that Cdh4 expressing tumor cells can form mainly heterophilic interactions with parenchymal cells which expresses only Cdh2. Cdh4-Cdh2heterophilic intercellular dimers (trans-heterodimers) are known to be weaker than respective homodimers [26,57,58]. Through its cytoplasmic tail, Cdh4 could also interferes with specific intracellular signaling pathways regulating cell proliferation and migration. Two of the main pathways dysregulated during the EMT process in malignant tumors are the Wnt and the Hippo pathways [3,59,60]. The lack of β-catenin in the nucleus of the analyzed control and Cdh4-silenced GIC cells suggests however that the canonical Wnt pathway is not activated in this context. A data analysis on microarray assay performed on GICs with different Cdh4 expression level does not highlight a differential expression level in genes involved in the Hippo pathway as *NF2*, *Lats1/2*, *Mst1*, *Yap1* (Dr. Antonio Daga, Ospedale Policlinico San Martino, Italy personal communication). Even if this data can not completely rule out a role of this signaling pathway in gliomagenesis, it is unlikely that Cdh4 can influence its activity. Further experiments showing the level of Hippo pathway activation by analyzing, for example, the phosphorylation level and the localization of the downstream effector YAP1 are necessary to completely clarify this issue.

Other two intracellular signaling molecules, key regulators of cell proliferation and migration, are ERK and AKT and often they result activated in cancer [61,62,63,64]. In our previous work on murine glioma cells we found a correlation between Cdh4 expression and ERK phosphorylation. In line with these results, we found in Cdh4-dowregulated GIC cells a reduction of the activated phosphorylated form of ERK respect to control GIC cells. This ERK inactivation can, at least in part, be responsible for the migratory and proliferative impairment of these cells.

## 4. Materials and Methods

### 4.1. Vectors

Six microRNA against Cdh4 mRNA (sequences are available on request) were designed using the BLOCK.iT RNAi Designer software of Life Technologies (version, Carlsbad, CA, USA). Each microRNA was separately cloned using the BLOCK.iT method into the pCDB-GW retroviral vector together with an EmGFP cassette as described previously [65]. The control vector is a pCAG:DsRed retroviral vector (kindly provided by Magdalena Goetz., Helmholtz Center, Munich, Germany).

Replication incompetent retroviral particles were obtained as previously described [66].

### 4.2. Glioma Initiating Cells Culture

Human GIC cultures derived by glioma patients (kindly provided by Antonio Daga, Ospedale Policlinico San Martino, Genova, Italy) were maintained on Matrigel coated flasks (1:200; BD Biosciences, San Jose, California, USA) in 50% Neurobasal, 50% DMEM/F12 media (Life Technologies, Paisley, UK), 1X B27 Supplement (Life Technologies), 10 ng/mL bFGF, 20 ng/mL EGF (Peprotech, London, UK), 2 mM glutamine (Life Technologies) and 2 µg/mL heparin (Sigma-Aldrich, Milano, Italy).

Control and Cdh4 microRNA transduced cultures were sorted thanks to DsRed or GFP expression on a FACSAria II (BD Biosciences) as previously described [67].

### 4.3. Animal Procedures

The in vivo experiments were approved by the Animal Ethics Committee (OPBA) of “Ospedale Policlinico San Martino” and by the Italian Ministry of Health (Authorization N° 859/2016-PR). All the experiments were performed according to the Italian law D. lgs 26/2014 and the European Directive 2010/63/EU of the European Parliament. In all the experiments were used the NOD.CB17-Prkdcscid/J strains (hereinafter referred as NOD/SCID) from ENVIGO (Huntingdon, UK).

Intracranial cell transplantation was performed as previously described [68]. Briefly, deeply anesthetized animals were mounted on a stereotaxic table and the skull was pierced with a 22G needle. By using a Hamilton Syringe, 4 × 10^5^ cells were injected at the following coordinates respect to the Bregma: 1 mm anterior; 1.5 mm lateral and 2.5 mm under the skull surface. At different time points, described in the Results section, brains were dissected and fixed over-night in 4% PFA at 4 °C and then cryoprotected in 20% sucrose over-night at 4 °C. Cryostat sections were stained with 1 µg/mL Hoechst-33342 (Sigma-Aldrich) for nuclei visualization.

### 4.4. Immunostaining

Cultured cells were usually fixed in 4% PFA for 15 min and washed three times in PBS. For Cdh2 staining, cells were fixed in methanol/acetone 1:1 at −20 °C for 10 min and washed as previously described. For the immunostaining, we used the following primary antibodies: Rabbit anti-Cdh2 (AbCam, Cambridge, UK); rat anti-Cdh4 (MRCD5, Hybridoma Bank, Iowa City, Iowa, USA); mouse anti-GM-130 (BD Biosciences); rabbit anti-β-catenin (Thermo Scientific, Waltham, Massachusetts, USA). We then used the following fluorescence secondary antibody from Jackson Immunoresearch Laboratories: Dylight 549- and Cy2-conjugated goat anti-mouse IgG, Dylight 549- and Dylight 488-conjugated goat anti-rabbit IgG, Cy3- and Alexa Fluor 488-conjugated goat anti-rat IgG. Nuclei were stained with 1 µg/mL Hoechst-33342 (Sigma-Aldrich).

### 4.5. Quantitative PCR and Western Blot

For quantitative PCR, cultured GICs were harvested in RLT lysis Buffer (Qiagen, Milano, Italy) and RNA was extracted following manufacturer instructions. cDNA was synthetized from 500 ng of RNA using the iScript Reverse Transcription Supermix (Bio-Rad) following the manufacturer’s instructions. One hundredth of the cDNA solution was used for q-PCR reaction using Luna Universal qPCR Master mix (AbCam). Cdh4 expression was normalized to the *Rpl41* housekeeping gene. The sequences of the primers are available on request.

For Western blot, cultured GICs were harvested in lysis buffer containing 50 mM HEPES (pH 7.5), 5 mM EDTA, 150 mM NaCl, 1% Triton X-100 detergent, and protease inhibitors (Complete, Roche Applied Science) and separated using Mini-Protean TGX gels (Bio-Rad, Segrate, Milano, Italy). Protein expression was normalized to α-tubulin. The following primary antibodies were used: Rat anti-Cdh4 (MRCD5, Hybridoma Bank, Iowa City, Iowa, USA), mouse monoclonal anti-α-tubulin (Sigma-Aldrich), rabbit anti-ERK1/2 (Cell Signaling, Danvers, Massachusetts, USA), rabbit anti-phosphoERK1/2 (Cell Signaling), rabbit anti-AKT (Cell Signaling), rabbit anti-phospho-AKT (Cell Signaling), rabbit anti p27 (Cell Signaling). Anti-mouse HRP-conjugated (Sigma-Aldrich), anti-rabbit HRP-conjugated (Sigma-Aldrich) and anti-rat HRP-conjugated (GE Healthcare, Little Chalfont, UK) secondary antibodies were used. Proteins were then revealed with the ECL Star substrate (Euroclone, Milano, Italy) and visualized with the MINI HD9 chemiluminescence system (Uvitec, Cambridge, UK). Quantification of the protein expression level was performed with the software ImageJ [69].

Data are reported as mean and standard errors of the mean.

### 4.6. Data Analysis

The software FIJI [70] was used for the analysis of the subcellular localization of β-catenin. Nuclear areas where identified by thresholding Hoechst signal. Perinuclear areas where defined as the 3 μm band surrounding the nuclear areas. Cytoplasmic/membrane-bound localization was inferred by subtracting nuclear and perinuclear from total cell area.

For the analysis of cell polarity, we used FIJI to obtain for each micrograph, the coordinates of cell nuclei, Golgi apparatuses and the rim of the scratch (details of the procedure are available on request). We then used a self-produced script in R [71], available on request, to measure, for each cell, the angle between the line joining the center of Golgi apparatus with the center of the nearest nucleus and the line perpendicular to the rim of the scratch. Cells with an angle between −60° and +60° were considered as polarized towards the scratch. Cells whose Golgi apparatus was not visible were not included in the analysis. Statistical analysis was performed by a Chi-squared test with two classes: Polarized cells (in null hypothesis expected to be one third of the total) and not-polarized cells (in null hypothesis expected to be two-third of the total).

For the in vivo analysis of the migratory ability of GICs transplanted cells, we used an Imager.M2 fluorescence microscope (version, ZEISS, Milano, Italy) controlled by the Slide Explorer plugin of μManager software (version v1.4, Ron Vale San Francisco California, USA) [72] to take 10× micrographs of brain sections around the injection sites. We then extracted, for each section, the coordinates of the needle track and of cell nuclei. With a self-produced script in R [71], available on request, we measured the distance of each GIC transplanted cell, detectable thanks to fluorescence reporter expression, from the rim of the injection site. Normalization between different brain sections were obtained by creating for each Section 10 bins for cell distance. We then measured the proportion of Cdh4 miRNA injected cells respect to control injected cells per each distance bin. To measure whether the imbalances in the cell frequencies depended on the cell distance from the injection site we used Pearson’s correlation test.

## 5. Conclusions

Overall our data demonstrated that Cdh4 could be a useful prognostic marker since its expression correlates with a shorter survival time for glioma patients. We demonstrated that Cdh4 contributes to high malignant features as the loss of cell–cell contact inhibition mechanisms that allows cells to proliferate undisturbed and to infiltrate the brain parenchyma. Moreover, our results showed that, although Cdh4 silenced glioma cells can proliferate and infiltrate the brain parenchyma, these abilities result impaired. It is worth to notice that in these experiments Cdh4 was not knocked-out but downregulated. Similar level of downregulation had a stronger effect in the mouse model, suggesting that in human glioblastomas another molecular mechanism can compensate its role.

In conclusion, we can affirm that Cdh4 is able to facilitate human glioblastoma cells to overcome CIP and CIM enhancing tumor proliferation and invasion.

## Supplementary Materials

Supplementary materials can be found at https://www.mdpi.com/1422-0067/20/16/4028/s1.

## Abbreviations

EMT	epithelial to mesenchymal transition
Cdh1	E-Cadherin
Cdh2	N-Cadherin
CIP	cell-cell inhibition of proliferation
CIM	cell-cell inhibition of migration
GIC	glioblastoma initiating cell
OPBA	Animal Ethics Committee
NOD/SCID	NOD.CB17-Prkdcscid/J
