# Supplementary figures and images for: Cdh4 Down-Regulation Impairs in Vivo Infiltration and Malignancy in Patients Derived Glioblastoma Cells
Source: Int J Mol Sci. 2019 Aug 18;20(16):4028. doi: 10.3390/ijms20164028 (PMC6718984; doi:10.3390/ijms20164028)

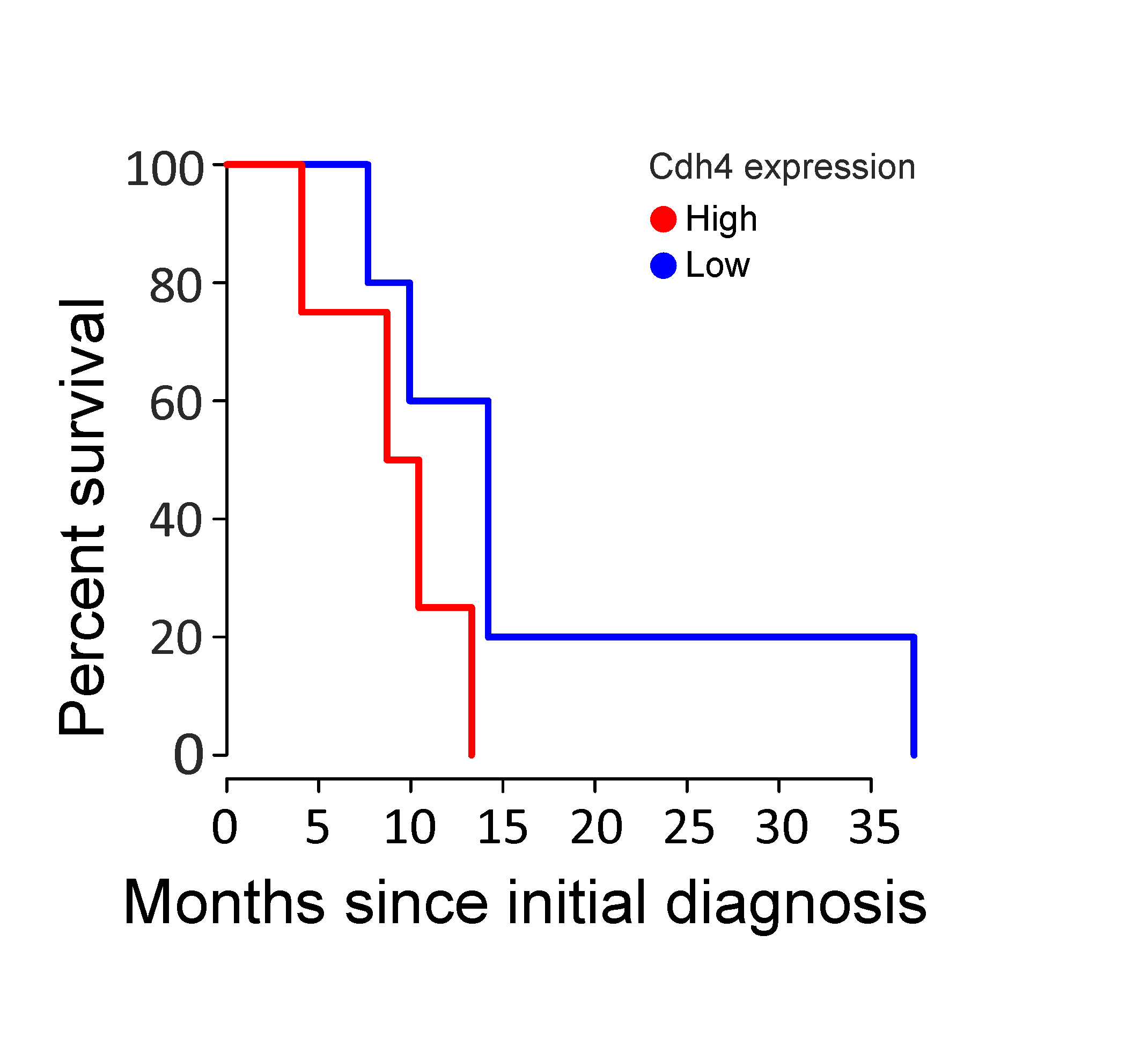

Supplement: Supplementary file 1 [file ijms-20-04028-s001.zip › Supplementary Figures S1-S2/Figure S1.tif]

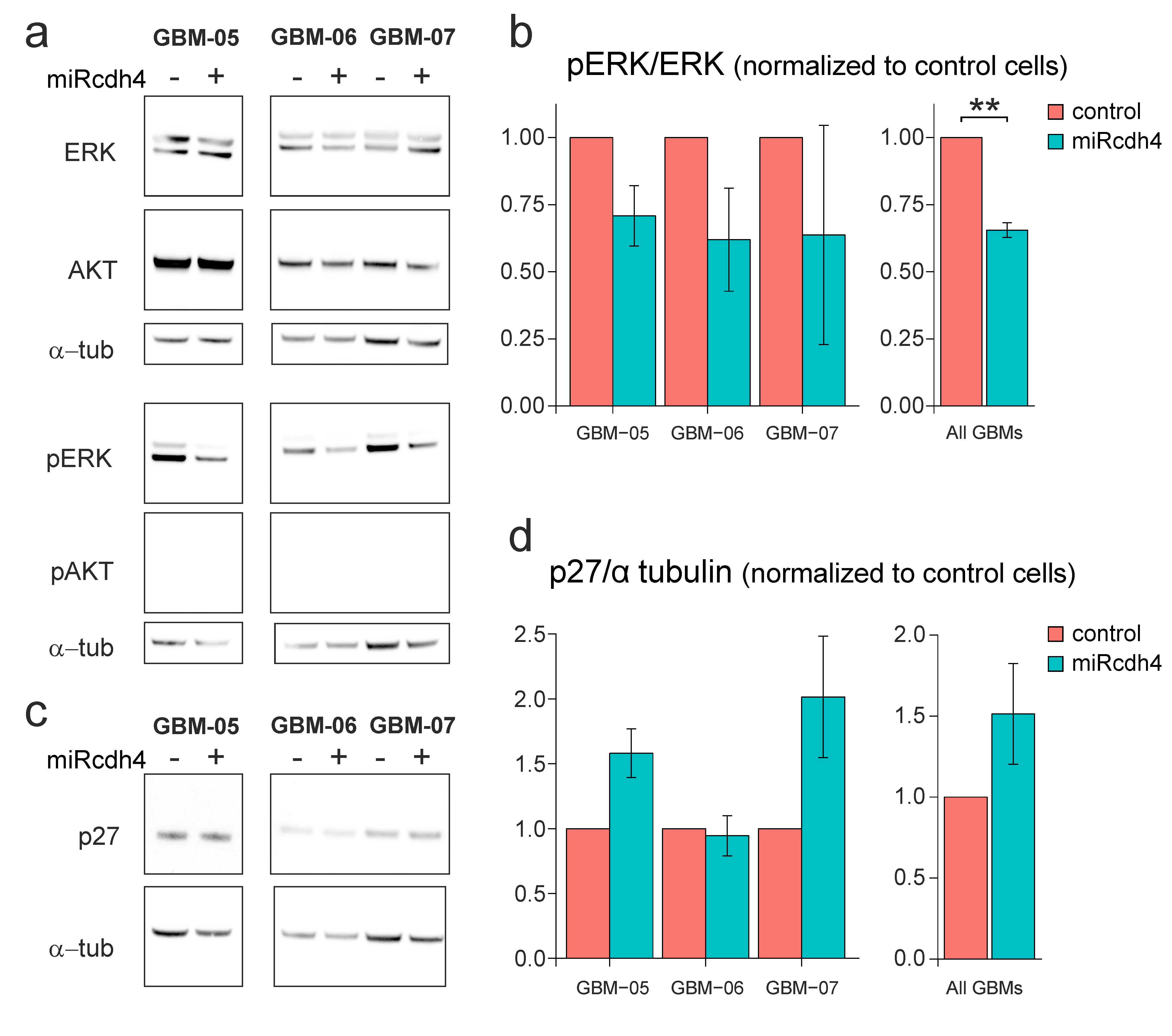

Supplement: Supplementary file 1 [file ijms-20-04028-s001.zip › Supplementary Figures S1-S2/Figure S2.tif]
